# Magnetic microscopy and topological stability of homochiral Néel domain walls in a Pt/Co/AlO_*x*_ trilayer

**DOI:** 10.1038/ncomms9957

**Published:** 2015-12-08

**Authors:** M. J. Benitez, A. Hrabec, A. P. Mihai, T. A. Moore, G. Burnell, D. McGrouther, C. H. Marrows, S. McVitie

**Affiliations:** 1 School of Physics and Astronomy, University of Glasgow, Glasgow G12 8QQ, Scotland; 2 School of Physics and Astronomy, University of Leeds, Leeds LS2 9JT, UK

## Abstract

The microscopic magnetization variation in magnetic domain walls in thin films is a crucial property when considering the torques driving their dynamic behaviour. For films possessing out-of-plane anisotropy normally the presence of Néel walls is not favoured due to magnetostatic considerations. However, they have the right structure to respond to the torques exerted by the spin Hall effect. Their existence is an indicator of the interfacial Dzyaloshinskii–Moriya interaction (DMI). Here we present direct imaging of Néel domain walls with a fixed chirality in device-ready Pt/Co/AlO_*x*_ films using Lorentz transmission electron and Kerr microscopies. It is shown that any independently nucleated pair of walls in our films form winding pairs when they meet that are difficult to annihilate with field, confirming that they all possess the same topological winding number. The latter is enforced by the DMI. The field required to annihilate these winding wall pairs is used to give a measure of the DMI strength. Such domain walls, which are robust against collisions with each other, are good candidates for dense data storage.

## Abstract (web summary)

Broken symmetry at the interface with a heavy metal gives rise to a chiral exchange interaction in ferromagnetic thin films, which may be used to control magnetic domain walls. Here, the authors demonstrate how this effect enforces topologically stable homochiral domain walls in a Pt/Co/AlO_x_ trilayer.

## Graphical abstract

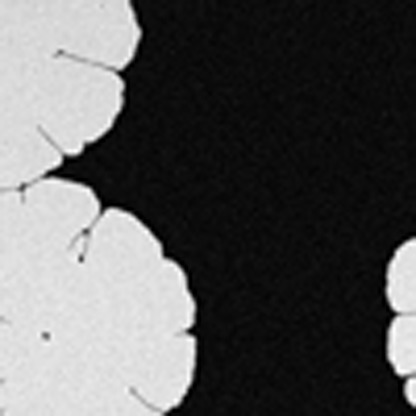

The broken inversion symmetry at interfaces between ferromagnets and heavy (high spin–orbit interaction) metals offers new ways to manipulate the magnetic state. The combination of a heavy metal and a thin ferromagnetic film gives rise to new phenomena which normally vanish in the bulk, but play an important role as soon as the thickness of the ferromagnet is reduced to an atomic scale. The presence of a Rashba electric field^1^ and Dzyaloshinskii–Moryia interaction (DMI)^2^ have been recently demonstrated in such systems. Therefore the classical picture of magnetism as being an interplay between exchange, dipolar and anisotropy energies is perturbed by a new energy term with very significant consequences. This DMI term is expressed as **D**·(**S**_*i*_ × **S**_*j*_), where **D** is the DMI vector, and **S**_*i*_ and **S**_*j*_ are spin moments sitting on neighbouring atoms. When **D** is sufficiently strong^3^, a non-uniform state has a lower energy than the ferromagnetic one, giving rise to chiral structures such as cycloids^4^, helices^5^ or skyrmions^6^,^7^ as the magnetic ground state. At an interface the DMI enforces spin textures of a cycloidal form^8^,^9^,^10^.

However, for small DMI values, domain walls (DWs) are the precursors of these non-uniform states. Here the DMI strength is imprinted into the static DW texture via a virtual effective magnetic field that prefers a Néel wall of a given chirality rather than the magnetostatically favoured Bloch wall^3^. It is important to note that this is only meaningful at the wall position since it is the direction of the wall, and its up–down or down–up character, that breaks the in-plane symmetry and defines the direction of this fictitious field. It has been shown that the choice of the heavy element dictates the sign and magnitude of the DMI^11^,^12^,^13^,^14^,^15^, selecting a particular sign ±1/2 of the soliton winding number (ref. 16) for every wall, defined as


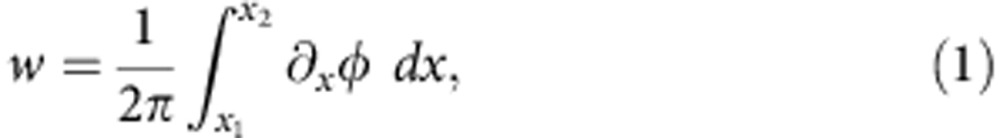


where *x* is a position co-ordinate across the wall, with *x*_1_ and *x*_2_ being positions deep inside the domains, and *φ* is the angle that the spin makes with the film normal. DWs are topological defects in the ferromagnetic state and may be classified by this winding number. The value of |*w*|=1/2 arises from the fact that the DW winds halfway round a circle in spin space, whilst the sign of *w* determines whether that winding is clockwise or anti-clockwise in direction. The detailed nature of the DW has remarkable consequences for the processes of DW dynamics^17^ and the sensitivity of the DW to the torques exerted on the localized magnetic moments^18^. The DW texture can be deduced indirectly by matching models with current^19^,^20^,^21^ and field-induced^3^,^12^,^22^,^23^ DW displacement data. DMI strength may also be measured using Brillouin light scattering^24^,^25^,^26^. Determination of the DW structure is therefore a key part of any investigation into these materials.

Historically, imaging of DWs in films with planar magnetization is well established, with many examples demonstrating the capability of imaging DWs which have widths upwards of tens to hundreds of nanometres. Methods such as magnetic-force microscopy^27^, photoemission electron microscopy^28^, scanning electron microscopy with polarization analysis^29^, electron holography^30^ or Lorentz transmission electron microscopy^31^ (L-TEM) have been used to image such DWs. The spatial extent of DWs in materials with out-of-plane anisotropy is often only 10 nm or less, making them interesting objects for high-density data storage devices. Resolving such small magnetic objects is a challenge: not all of these methods have the required spatial resolution. Spin-polarized scanning tunnelling microscopy^32^ and spin-polarized low-energy electron microscopy^13^ manifested the Néel texture of the walls in bilayer systems grown and studied *in situ*, while nitrogen-vacancy microscopy revealed and demonstrated the difference of the stray field distribution above the Bloch and Néel walls in trilayer systems such as Pt/Co/AlO_*x*_ or Pt/Co/Pt^33^. Pt/Co/AlO_*x*_ is a prototype heavy metal/ferromagnet/oxide system of the sort that is extremely interesting for solid-state memory devices^34^ owing to the combination of high DW velocities^35^ (with various phenomena being proposed to explain this process^1^,^36^,^37^) with the oxide layer which can be used as a tunnel barrier for the information read-out. Here we use L-TEM and Kerr microscopy to directly image DWs in perpendicularly magnetized device-ready films of Pt/Co/AlO_*x*_, allowing us to deduce the presence of narrow homochiral—that is, possessing the same soliton winding number—Néel walls. Furthermore, measurements taken using both L-TEM and polar Kerr imaging at ambient temperature, which demonstrate the robustness against field owing to the mutual topological winding of these walls when they are forced together by a field^38^,^39^, are consistent with the presence of a measurable DMI^40^.

## Results

### Néel walls

**Figure 1:**
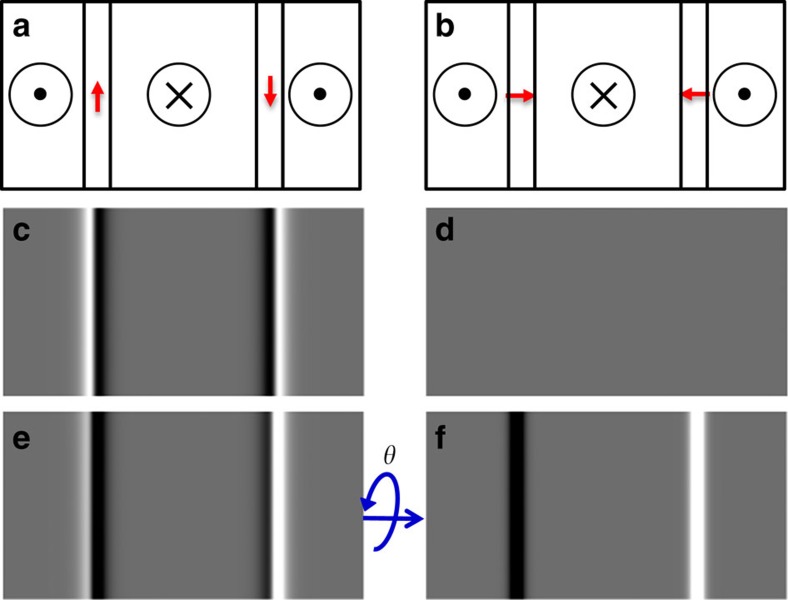
Calculated contrast for Bloch and Néel domain walls. Top view sketch of Bloch (**a**) and Néel (**b**) walls in a thin film. The red arrows show the direction of the moments in the centre of the wall between the domains with indicated magnetization orientation. The calculated defocused Fresnel L-TEM contrasts for these DWs are shown in **c** and **d** for cases with a sample tilt of *θ*=0° away from normal incidence, respectively. (**e**,**f**) Calculated contrast for *θ*=30° where the blue arrows indicate the sample tilt axis and its rotation direction. The dark or white electron beam intensity contrasts correspond to regions where the magnetic induction distribution results in the electron beam converging or diverging, respectively.

The two main types of DWs for films possessing out-of-plane anisotropy, Bloch and Néel, are sketched in Fig. 1a,b in top view. The magnetization within the Bloch wall does not produce any magnetic poles apart from the positions where they intersect the surfaces of the film, which are well separated for walls of any significant length. In contrast to this, the Néel wall gives rise to positive and negative magnetic charges along the whole length of the DW, separated by the DW width Δ, which is of the order of Δ≅(*A*/*K*)^1/2^, where *A* is the exchange stiffness and *K* is the effective anisotropy constant. The wall width Δ is typically several nanometres in this sort of system. Thus the Bloch wall is energetically favoured in perpendicularly magnetized thin films on magnetostatic grounds. To rotate the magnetic moments from the Bloch into the Néel configuration, an in-plane anisotropy, expressed as *K*_D_=*N*_*x*_*μ*_0_*M*_s_^2^/2, where *N*_*x*_ is the demagnetizing factor^41^ that depends on the DW width Δ and film thickness *t*, has to be overcome. This can be achieved either by applying an external magnetic field in the film plane to lift the DW out of its Bloch ground state^36^, or, for suitable inversion symmetry-breaking over and underlayers, the ground state can be changed to Néel by a fictitious magnetic field representing the effects of the DMI^3^. Figure 1 shows pairs of walls that both wind in the same direction, so *w*, defined in equation 1, has the same sign for both walls. The other winding direction is also possible: for DMI-free Bloch DWs the two are energetically equivalent, whilst the sign of the DMI that generates the Néel wall type energetically favours a particular handedness of winding.

In order to observe the DWs, Fresnel-mode L-TEM imaging was carried out, which reveals DWs as lines of black/white contrast^42^. For materials with in-plane magnetization this is achieved by defocussing the imaging lens; the induction either side of the DW results in a Lorentz deflection and the electron beam diverges or converges at the wall giving dark- or bright-wall contrast. However, for perpendicularly magnetized films, no deflection can arise from the out-of-plane magnetization component if the electron beam is at normal incidence. Thus any contrast will arise from the in-plane component, which only exists at the position of the DW. For the case of Bloch and Néel DWs, the calculated Fresnel images are shown in Fig. 1c and d for normal incidence. The Bloch wall shows black/white contrast, as has been observed experimentally^43^, since each edge of the DW is similar to diverging or converging wall with no induction on one side. In the case of Néel walls at normal incidence no contrast is visible, as shown in Fig. 1d. The basis of contrast in Lorentz microscopy is that the magnetization configuration must have a component of magnetization curl parallel to the electron beam direction^44^; there is no such component present in the Néel wall. This distinguishes the two walls, and means the Néel walls are invisible in this sample orientation.

The Néel wall can be made visible by tilting the film along an axis perpendicular to the wall in the plane of the film so that the electron beam no longer passes through the film at normal incidence. This then results in a component of magnetization/induction in the domains that is perpendicular to the beam and gives only black or only white contrast at the walls, as shown in Fig. 1f. Tilting of a film with a Bloch wall results in contrast observed in Fig. 1e, which retains its black/white character although now in an asymmetric form. In this way the wall positions are identified and their forms distinguished.

**Figure 2:**
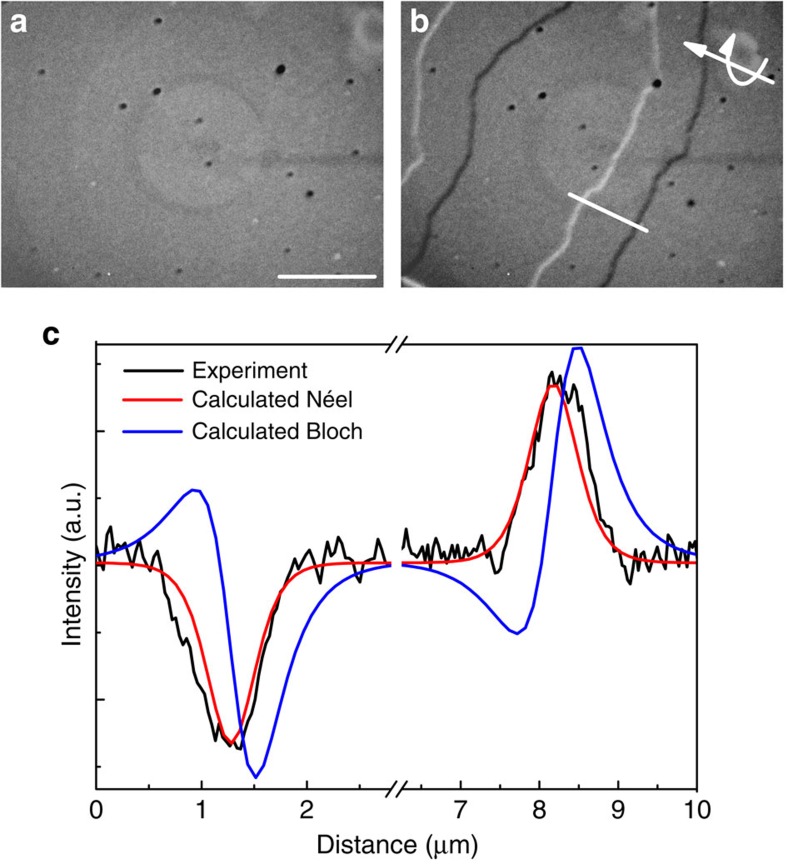
Fresnel-mode L-TEM images of the Pt/Co/AlO_*x*_ sample. (**a**) At normal incidence, *θ*=0°, the DWs generate no contrast and are invisible. Scale bar, 10 μm. (**b**) For a sample tilted around the rotation axis indicated by the white arrows by *θ*=30°, the DWs appear. That the same area has been imaged is confirmed by the pattern of dark spots, which correspond to defects or dust particles. (**c**) Contrast linetraces: the experimental contrast was obtained at the position of the white line in **b**. The calculated Bloch and Néel wall contrast was extracted from Fig. 1c. Note that the apparent wall width is far higher than the actual wall width owing to the convolution of the wall profile with the resolution of the Lorentz-defocussed microscope.

The walls under investigation here in sputtered Pt/Co/AlO_*x*_ trilayers were imaged in the Fresnel mode, and the representative results are shown in Fig. 2. First, the film was subjected to an out-of-plane applied field of ∼8 mT using the objective lens, close to the value of the coercivity, to induce DWs. After the applied field had been removed, Fresnel images were taken both untilted and tilted by 30°, which are shown in Fig. 2a,b, respectively. It is apparent that no DWs at all are visible when the film is untilted, furthermore when tilted the DW contrast appears either dark or bright without any dark/bright asymmetry. An intensity linetrace across bright and dark walls is shown in Fig. 2c confirming the symmetric nature of the contrast. (A small-intensity variation across the image can be seen in both images which is non-magnetic in origin). To illustrate the difference between Bloch and Néel walls, calculated intensity variations, derived from the tilted images in Fig. 1e,f and based on realistic wall widths (Δ≅10 nm, see Methods) and the known imaging conditions when the microscope is defocussed, are shown in Fig. 2c for comparison with the data. The clear asymmetry expected from the Bloch walls is not visible in the experimental linetrace. We conclude therefore that the walls are of the Néel type. It should be noted that the component of the magnetization at the centre of the Néel wall gives no contrast in Fresnel images in this configuration. Therefore, whilst we can conclude that these are indeed Néel walls, it is not possible to say anything about the chirality (the sign of *w*) from such images alone.

### Homochiral walls

**Figure 3:**
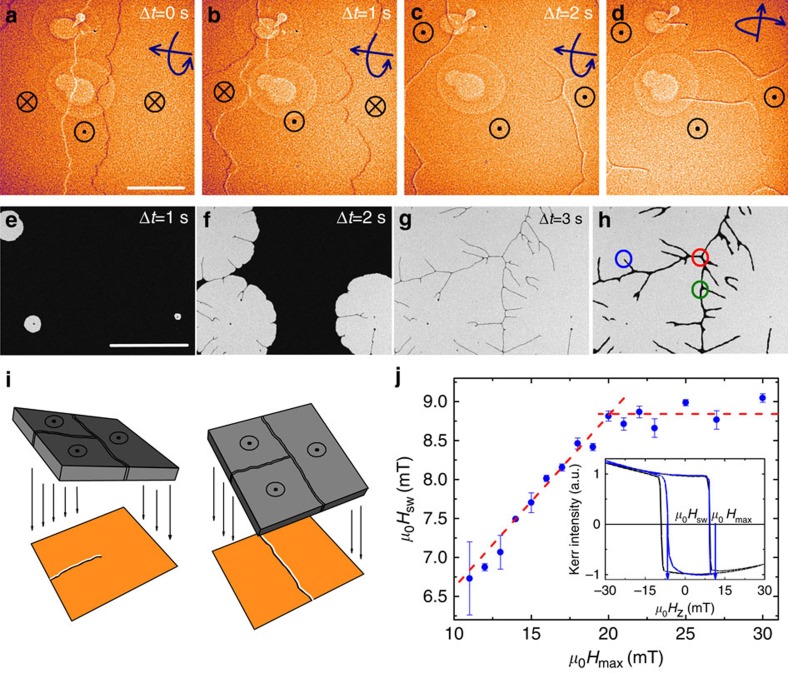
Field-driven DW displacement experiments. (**a**–**c**) L-TEM images of the DWs displaced by external field of *μ*_0_*H*_ext_=2 mT as a function of time. Symbols denote orientation of the magnetization. Scale bar, 20 μm. (**d**) The character of the Néel walls is confirmed by tilting the sample as indicated by the arrows. (**e**–**g**) Polar Kerr microscopy images showing the magnetization reversal process, with topologically protected 360° DW structures indicated by the black lines between domains at 7 mT (**g**). Therefore they behave as artificial nucleation centres when the polarity of the external magnetic field is reversed in **h**. Here the free end of the DW loop is marked with a blue circle, whilst the triple points are marked with green and red circles. (**i**) The contrast from two perpendicular DWs can be revealed by tilting the sample with the respect to the DW orientation. Scale bar, 100 μm. (**j**) Switching field *μ*_0_*H*_sw_ as a function of *μ*_0_*H*_max_ where the dashed lines correspond to the two linear regimes. Inset shows hysteresis loop cycled between ±30 mT (black) and +30 mT and *μ*_0_*H*_max_ (blue). A total of, 10 hysteresis loops were averaged to determine *μ*_0_*H*_sw_.

An additional micromagnetic energy term, beyond the usual exchange, anisotropy and magnetostatic contributions, is required in order to explain the presence of Néel, rather than Bloch walls. At present the only known candidate is the interfacial DMI^3^,^8^, in which case all the walls have the same handedness of winding (same sign of *w*) owing to the DMI's chirality selecting effect. In order to confirm this hypothesis of unique DW chirality, a vertical magnetic field was applied in the TEM to bring the DWs together to observe the subsequent interaction between them. Figure 3a–c shows a sequence of images of DWs displaced by a magnetic field. The initial magnetic state shown in Fig. 3a was imaged before the magnetic field is applied. Figure 3b,c show the magnetic state during and after the application of a magnetic field *μ*_0_*H*_ext_=2 mT. Figure 3d was acquired under the same conditions as Fig. 3c, but for a different tilt direction as depicted schematically in Fig. 3i. The appearance of walls only along the tilt direction (see Fig. 3i) confirms their Néel state. It can be seen that the up-domain state grows and the down-domain shrinks until the point where the two DWs meet, at which point they merge into 360° DWs^39^. A much larger magnetic field has to be applied in order to annihilate these and fully saturate the film. The formation of 360° DWs takes place when two DWs of the same winding number meet—a so-called ‘winding pair'^45^—and are unable to annihilate, since an energy barrier must be overcome when the wall core rotates out of the easy direction defined by the combination of anisotropy and DMI. This energy barrier is not present when the walls have opposite winding number and form an unwinding pair, the magnetostatic charges of which are attractive, meaning that such walls will collide and annihilate spontaneously when brought into close enough proximity.

Winding pairs can form by chance in a chirally degenerate system. When two 180° walls collide, in this case the probability that both walls have the same sign of winding number, so that Σ*w*=±1 for the pair, is 1/2. These walls will form hard-to-annihilate 360° winding pairs. On the other hand, the probability of two members of the pair possessing opposite *w*, so Σ*w*=0 equivalent to the ferromagnetic state, is also 1/2. These pairs can easily unwind owing to their mutual magnetostatic attraction and absence of any energy barrier^38^,^45^. We emphasize that all of the *N*>10 DW pairs we observed formed 360° DWs when they met, showing that these Néel walls are homochiral, a situation that is enforced by the presence of an interfacial DMI. The probability of this happening by chance in a system where there is no DMI to enforce chirality is 1/2^*N*^<0.1%, small enough to ignore. Thus we can conclude that our system is, in fact, homochiral.

### DMI strength

A sufficiently strong magnetic field must be applied to overcome the energy barrier and annihilate the 360° DW. Measuring the field required to do this has been suggested as a means of experimentally determining the strength of the DMI^40^. To observe a meaningful number of annihilation events, we have mimicked the L-TEM experiment with Kerr microscopy measurements in which the up and down domains are easily visible as dark or bright contrast. After saturation, reverse domains nucleate at point defects and expand as bubbles until they meet each other, whereupon the pairs of walls stabilize into a Voronoi-like network of 360° wall structures, with one cell surrounding each nucleation point. Domain expansion upon the application of a magnetic field during this process is shown in the Kerr micrographs in Fig. 3e–g and also in Supplementary Movie 1, exhibiting the same generic behaviour that was observed by the L-TEM. The black lines appearing in Fig. 3g correspond to the two Néel walls squeezed together into a 360° structure. Since the magnetic field is not large enough to annihilate them, it is possible to pull them apart again by applying a small magnetic field of the opposite polarity as depicted in Fig. 3h, preserving the topology of the Voronoi-like network. The coercive field *μ*_0_*H*_c_=9.0±0.2 mT can be extracted from the Kerr effect hysteresis loop shown in the inset in Fig. 3j, measured with the magnetic field swept in the range ±30 mT.

In order to measure the annihilation field, the following magnetic field sequence, illustrated by the blue curve in the inset of Fig. 3j, was applied. The film was first saturated at −30 mT and then the magnetic field was ramped to a maximum field *μ*_0_*H*_max_ above the coercive field. The magnetic field is then swept back to −30 mT and so the switching field *μ*_0_*H*_sw_ can be measured. It is clear from the Fig. 3j inset that the film now switches at a field that is lower than the coercive field of the major loop, i.e., *μ*_0_*H*_sw_≤*μ*_0_*H*_c_. In this case, the nucleation process is qualitatively different to that after full saturation has been achieved. Growth of reverse domains starts from the network of unannihilated winding DW pairs, as shown in Supplementary Movie 2.

Figure 3j shows the dependence of the switching field *μ*_0_*H*_sw_ on the maximum field *μ*_0_*H*_max_. The switching field rises linearly until it is as large as *μ*_0_*H*_c_, which occurs at *μ*_0_*H*_max_=20.0±0.5 mT. For *μ*_0_*H*_max_ larger than this, *μ*_0_*H*_sw_=*μ*_0_*H*_c_. The linear rise is caused by a greater proportion of annihilated 360° walls, which leads to a lower number of pre-nucleated regions for subsequent reverse domain growth on the reverse portion of the field sweep and a higher switching field. (This is the reason that DWs do not nucleate at a complete Voronoi-like network in Supplementary Movie 2, some parts of the network have been annihilated.) Once all the DWs are annihilated at *μ*_0_*H*_ann_≥20.0±0.5 mT, the reversal process is governed by reversed domain nucleation and propagation—just as on the major loop—rather than propagation alone. Since the stability of the two Néel walls in the 360° structure depends on the magnitude of the DMI, the annihilation field of two homochiral walls is a direct evaluation of the DMI^40^.

**Figure 4:**
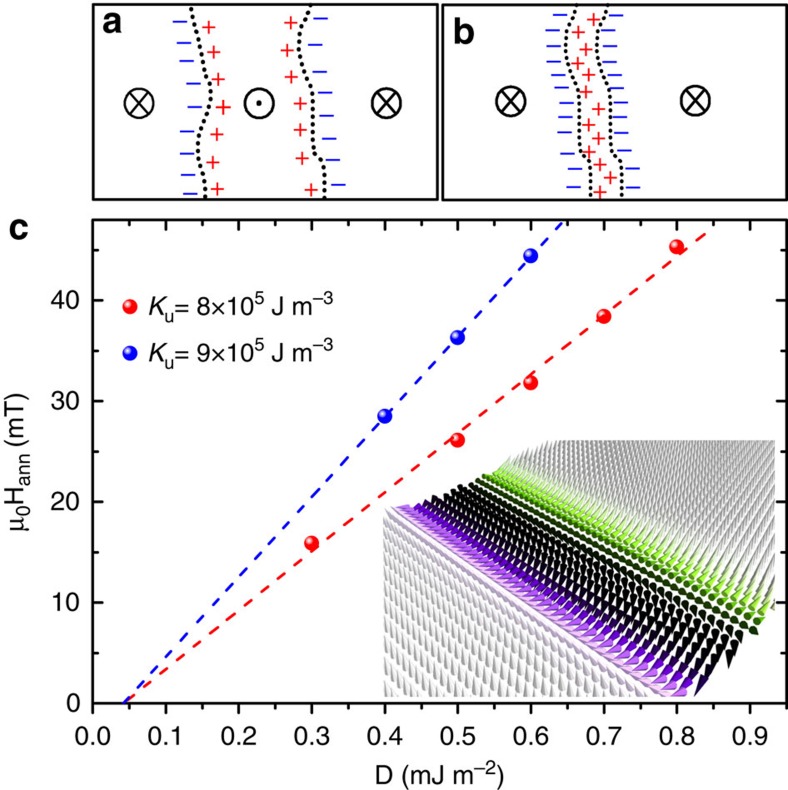
Topological prevention of domain wall annihilation by homochirality. (**a**) Two 180° Néel DWs with the same chirality in down–up–down magnetized film, showing the magnetostatic charges. (**b**) A composite 360° Néel DW after application of a small out-of-plane magnetic field that drives them together. The magnetostatic charge prevents the two DWs from annihilating. (**c**) DW annihilation field as a function of *D* as calculated by micromagnetic simulations for different uniaxial anisotropy values. The inset illustrates the simulated magnetization profile of the up–down–up domain including two Néel walls squeezed together by an external field. Only a small section of a much larger film is depicted in this image.

Micromagnetic simulations were performed to evaluate the effect of the DMI on the DW annihilation field. To investigate this problem we first prepared two DWs in a simulated nanowire of lateral dimensions 256 nm × 1,024 nm by introducing an up–down–up domain structure. Periodic boundary conditions were used to prevent the nucelation of topological defects at the edges of the material. The two DWs were set to be 300 nm apart and then brought together by an external field as shown in the inset of Fig. 4c. Figure 4c shows the calculated dependence of the annihilation field on *D* for different anisotropies to include the error of the anisotropy measurement. We find that our measured value of *μ*_0_*H*_ann_ implies that |*D*|=0.33±0.05 mJ m^−2^. The micromagnetic simulations were performed in defect-free material and at *T*=0 K, and since there is a possibility of DW annihilation due to thermal activation, this value is only a lower limit of *D*. The real *D* could be much higher, potentially reaching similar value *D*≅2.2 mJ m^−2^ as measured by Pizzini *et al.*^37^. In any case, the most pessimistic value of |*D*|=0.33±0.05 mJ m^−2^ is already high enough to satisfy the condition *D*>2*N*_*x*_*μ*_0_*M*_s_^2^Δ/*π* needed to enforce the Néel configuration for the DWs in this system^3^.

### Topological considerations

The network of walls also has its own topology. The pattern of walls is not a perfect Voronoi network since the walls are not perfectly straight and there are some free branches, for example, that marked with a blue circle in Fig. 3h. These free DW branches are a consequence of individual 180° DWs being pinned at imperfections: the bubble continues to expand leaving the 180° DW wrapped around it. This process can be clearly seen in Supplementary Movies 1 and 2. Once the bubbles are all maximally expanded, this (winding) pair of walls meets the bubble edge at a triple point, with an example marked by a green circle in Fig. 3h. Other triple points are formed at the vertices of the Voronoi-like network: an example is marked with a red circle in Fig. 3h. The first kind of triple point is distinct from the second in that removal of the pinning defect and absorption of the free end into the wall will not change the overall topology of the network, and we counted only independently nucleated wall pairs, and not these branches with a free end pinned at a defect, when counting the number of pairs *N* which form 360° winding pairs upon meeting.

In the foregoing we have used one-dimensional soliton winding numbers, defined by equation 1, to discuss the properties of the DWs. If the spins are confined to a plane (so that spin space is two-dimensional) and we integrate along the line *x*_1_→*x*_2_ then we are concerned with the homotopy group *π*_1_(*S*^1^) and there is topological protection of pairs of winding walls in the strongest sense: there is no way to smoothly deform a pair of winding walls, with Σ*w*=1, into the uniformly magnetized state, where Σ*w*=0. (The usual caveats about edges and the fact that real materials have a discrete atomic lattice need to be borne in mind.) Nevertheless, this does not properly describe the reality of the situation: in fact we have Heisenberg spins, and the spin space is the two-sphere *S*^2^. Here we have topological protection in a weaker sense^16^,^46^, in that, whilst smooth deformation to the ferromagnetic state is possible, the relationship between the walls solitonic topological winding numbers determines whether or not the walls must cross an energy barrier to do so. Thus mutual unwinding is impossible unless the field is large enough. This picture usefully underpins all of the foregoing discussion.

However, we have also so far neglected the fact that we have a two-dimensional system. The relevant homotopy group is *π*_2_(*S*^2^) and, strictly speaking, when considering topological stability we ought to make use of the skyrmion winding number, defined by ref. 16


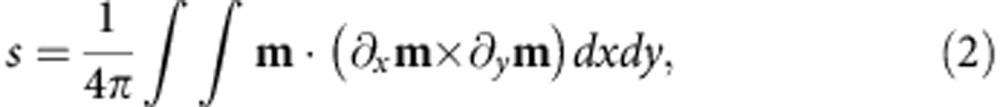


where **m** is a unit vector in the direction of the local magnetization. Neither a Bloch or a Néel wall possesses a non-zero value of *s* (and so exhibits strong topological protection) unless it is wrapped into a closed loop. When the area inside such a loop shrinks to a point it is known as a skyrmion^47^.

Since our system is homochiral each bubble domain that nucleates is topologically equivalent to a skyrmion^48^, with *s*=±1, since the DMI enforces a given wall chirality all around its perimeter. The sign of *s* is the same for all bubbles and is set by the DMI sign. As a result, each triple point inherits a skyrmion winding number 
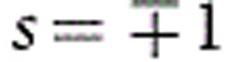
 from the bubbles that form it, since spins can be found in the vicinity of each triple point that completely wrap the 2-sphere *S*^2^. One might therefore expect that there are triple points which are especially stable against field. We have checked that these structures do not contribute to the annihilation field with a further micromagnetic simulation incorporating an up–down–up DW structure in two dimensions and thus an *s*=1 point at the centre of a square element. For the same input micromagnetic parameters *A*, *D*, *K* and *M*_s_ the annihilation field is barely affected.

Our L-TEM experiments show that DWs in the Pt/Co/AlO_*x*_ system are indeed of the Néel form, enforced by an interfacial DMI overcoming the preference of the magnetostatic energy term for Bloch walls. The fact that they always form 360° structures when pressed together by a field shows that these walls are homochiral, just as is expected in the presence of a DMI. We have implemented the method of annihilating these 360° structures with a field in order to estimate the strength of the DMI in this system as being at least |*D*|=0.33±0.05 mJ m^−2^. This confirms the widespread assumption, made to interpret field- or current-driven DW motion data, that such walls are of the Néel type owing to an effective field arising from the interfacial DMI. Knowledge of wall structures is not only important in DW dynamics experiments and the design of spintronic technologies based on this phenomenon, such as racetrack memories, where the fact that the DWs are topologically protected from mutual annihilation means that they can be very closely packed, permitting data density. Also, these Néel type walls, wrapped in a circle, form the boundaries of bubble domains with non-zero skyrmion winding numbers^48^, leading to inertial dynamics^49^ and with potential for use in skyrmion-based spintronic devices^50^,^51^.

## Methods

### Sample preparation

Multilayer films with an active trilayer of Pt(3 nm)/Co(0.8 nm)/AlO_*x*_(3 nm) were deposited on a Ta (3.2nm) buffer by sputtering at base pressure 10^−8^ Torr. These layered films were supported on bulk Si substrates for magneto-optic Kerr effect measurement and 35 nm thick amorphous Si_3_N_4_ membranes for TEM observation. The Pt and Co layers were deposited using dc sputtering, whilst the AlO_*x*_ layer was deposited by rf sputtering from an oxide target. Si_4_N_3_ membranes were used as a substrate, meaning that the samples were immediately ready for transmission electron microscopy studies. The effective anisotropy constant *K* was measured using a superconducting quantum interference device vibrating sample magnetometer (SQUID-VSM).

### Magnetization imaging

The L-TEM images shown here were acquired using an FEI Tecnai T20 TEM operated at 200 kV in Lorentz mode, with the objective lens only weakly excited^52^. Kerr imaging was carried out in an Evico microscope. The perpendicular magnetic anisotropy of the deposited films was confirmed by polar Kerr microscopy showing square hysteresis loops (for example, that in Fig. 3i).

### Micromagnetic calculations

Micromagnetic modelling was carried out using the MuMax3 code^53^. The cell size used was 1 nm × 1 nm × 0.8 nm. In this model we used the following material parameters appropriate to Co (ref. 54): saturation magnetization *M*_s_=1.1 × 10^6^ A m^−1^, perpendicular uniaxial anisotropy *K*_u_=9±1 × 10^5^ J m^−3^ (both measured by SQUID-VSM) and exchange stiffness *A*=16 pJ m^−1^.

### Data availability

All relevant data present in this publication can be accessed at http://dx.doi.org/10.5525/gla.researchdata.217.

## Additional information

**How to cite this article:** Benitez, M. J. *et al.* Magnetic microscopy and topological stability of homochiral Néel domain walls in a Pt/Co/AlO_*x*_ trilayer. *Nat. Commun.* 6:8957 doi: 10.1038/ncomms9957 (2015).

## Supplementary Material

Supplementary Movie 1 Kerr microscopy of reversed domain nucleation and demonstration of the domain wall protection

Supplementary Movie 2 Kerr microscopy of magnetization reversal starting from non-annihilated domain walls.

## References

[b1] Miron I. M. *et al.* Current-driven spin torque induced by the Rashba effect in a ferromagnetic metal layer. Nat. Mater. 9, 230–234 (2010). 20062047 10.1038/nmat2613

[b2] Heide M., Bihlmayer G. & Blügel S. Dzyaloshinskii-moriya interaction accounting for the orientation of magnetic domains in ultrathin films: Fe/W(110). Phys. Rev. B 78, 140403 (2008).

[b3] Thiaville A., Rohart S., Jué E., Cros V. & Fert A. Dynamics of Dzyaloshinskii domain walls in ultrathin magnetic films. Europhys. Lett. 100, 57002 (2012).

[b4] Ferriani P. *et al.* Atomic-scale spin spiral with a unique rotational sense: Mn monolayer on W (001). Phys. Rev. Lett. 101, 027201 (2008). 18764220 10.1103/PhysRevLett.101.027201

[b5] Uchida M., Onose Y., Matsui Y. & Tokura Y. Real-space observation of helical spin order. Science 311, 359–361 (2006). 16424334 10.1126/science.1120639

[b6] Heinze S. *et al.* Spontaneous atomic-scale magnetic skyrmion lattice in two dimensions. Nat. Phys. 7, 713–718 (2011).

[b7] Chen G., Mascaraque A., N'Diaye A. T. & Schmid A. K. Room temperature skyrmion ground state stabilized through interlayer exchange coupling. Appl. Phys. Lett. 106, 242404 (2015).

[b8] Fert A. Magnetic and transport properties of metallic multilayers. Mater. Sci. Forum 59-60, 439–480 (1991).

[b9] Crépieux A. & Lacroix C. Dzyaloshinsky-Moriya interactions induced by symmetry breaking at a surface. J. Magn. Magn. Mater. 182, 341–349 (1998).

[b10] Freimuth F., Blügel S. & Mokrousov Y. Berry phase theory of Dzyaloshinskii-Moriya interaction and spin-orbit torques. J. Phys. Condens. Matter 26, 104202 (2014). 24552898 10.1088/0953-8984/26/10/104202

[b11] Ryu K.-S., Yang S.-H., Thomas L. & Parkin S. S. P. Chiral spin torque arising from proximity-induced magnetization. Nat. Commun. 5, 3910 (2014). 24852680 10.1038/ncomms4910

[b12] Hrabec A. *et al.* Measuring and tailoring the Dzyaloshinskii-Moriya interaction in perpendicularly magnetized thin films. Phys. Rev. B 90, 020402 (2014).

[b13] Chen G. *et al.* Tailoring the chirality of magnetic domain walls by interface engineering. Nat. Commun. 4, 2671 (2013). 24154595 10.1038/ncomms3671

[b14] Torrejon J. *et al.* Interface control of the magnetic chirality in CoFeB/MgO heterostructures with heavy metal underlayers. Nat. Commun. 5, 4655 (2014). 25130480 10.1038/ncomms5655

[b15] Chen G. *et al.* Novel chiral magnetic domain wall structure in Fe/Ni/Cu (001) films. Phys. Rev. Lett. 110, 177204 (2013). 23679766 10.1103/PhysRevLett.110.177204

[b16] Braun H.-B. Topological effects in nanomagnetism: from superparamagnetism to chiral quantum solitons. Adv. Phys. 61, 1–116 (2012).

[b17] De Ranieri E. *et al.* Piezoelectric control of the mobility of a domain wall driven by adiabatic and non-adiabatic torques. Nat. Mater. 12, 808–814 (2013). 23749266 10.1038/nmat3657

[b18] Khvalkovskiy A. *et al.* Matching domain-wall configuration and spin-orbit torques for efficient domain-wall motion. Phys. Rev. B 87, 020402 (2013).

[b19] Emori S., Bauer U., Ahn S.-M., Martinez E. & Beach G. Current-driven dynamics of chiral ferromagnetic domain walls. Nat. Mater. 12, 611–616 (2013). 23770726 10.1038/nmat3675

[b20] Ryu K.-S., Thomas L., Yang S.-H. & Parkin S. S. P. Chiral spin torque at magnetic domain walls. Nature Nano 8, 527–533 (2013). 10.1038/nnano.2013.102 23770808

[b21] Lo Conte R. *et al.* Role of B diffusion in the interfacial Dzyaloshinskii-Moriya interaction in Ta/Co_20_Fe_60_B_20_/MgO nanowires. Phys. Rev. B 91, 014433 (2015).

[b22] Je S.-G. *et al.* Asymmetric magnetic domain-wall motion by the Dzyaloshinskii-Moriya interaction. Phys. Rev. B 88, 214401 (2013).

[b23] Kim D.-Y., Kim D.-H., Moon J. & Choe S.-B. Determination of magnetic domain-wall types using Dzyaloshinskii–Moriya-interaction-induced domain patterns. Appl. Phys. Lett. 106, 262403 (2015).

[b24] Nembach H. T., Shaw J. M., Weiler M., Jué E. & Silva T. J. Linear relation between Heisenberg exchange and interfacial Dzyaloshinskii-Moriya interaction in metal films. Nat. Phys. 11, 825–829 (2015).

[b25] Belmeguenai M. *et al.* Interfacial Dzyaloshinskii-Moriya interaction in perpendicularly magnetized Pt/Co/AlO_*x*_ ultrathin films measured by Brillouin light spectroscopy. Phys. Rev. B 91, 180405 (2015).

[b26] Cho J. *et al.* Thickness dependence of the interfacial Dzyaloshinskii-Moriya interaction in inversion symmetry broken systems. Nat. Commun. 6, 7635 (2015). 26154986 10.1038/ncomms8635 PMC4510697

[b27] Yamaguchi A. *et al.* Real-space observation of current-driven domain wall motion in submicron magnetic wires. Phys. Rev. Lett. 92, 077205 (2004). 14995881 10.1103/PhysRevLett.92.077205

[b28] Kläui M. *et al.* Head-to-head domain-wall phase diagram in mesoscopic ring magnets. Appl. Phys. Lett. 85, 5637–5639 (2004).

[b29] Lepadatu S., Vanhaverbeke A., Atkinson D., Allenspach R. & Marrows C. H. Dependence of domain-wall depinning threshold current on pinning profile. Phys. Rev. Lett. 102, 127203 (2009). 19392318 10.1103/PhysRevLett.102.127203

[b30] Backes D. *et al.* Transverse domain walls in nanoconstrictions. Appl. Phys. Lett. 91, 112502 (2007).

[b31] Basith M., McVitie S., McGrouther D., Chapman J. & Weaver J. Direct comparison of domain wall behavior in permalloy nanowires patterned by electron beam lithography and focused ion beam milling. J. Appl. Phys. 110, 083904 (2011).

[b32] Kubetzka A., Bode M., Pietzsch O. & Wiesendanger R. Spin-polarized scanning tunneling microscopy with antiferromagnetic probe tips. Phys. Rev. Lett. 88, 057201 (2002). 11863771 10.1103/PhysRevLett.88.057201

[b33] Tetienne J.-P. *et al.* The nature of domain walls in ultrathin ferromagnets revealed by scanning nanomagnetometry. Nat. Commun. 6, 6733 (2015). 25828294 10.1038/ncomms7733

[b34] Miron I. M. *et al.* Perpendicular switching of a single ferromagnetic layer induced by in-plane current injection. Nature 476, 189–193 (2011). 21804568 10.1038/nature10309

[b35] Moore T. A. *et al.* High domain wall velocities induced by current in ultrathin Pt/Co/AlO_x_ wires with perpendicular magnetic anisotropy. Appl. Phys. Lett. 93, 262504 (2008) Erratum **95**, 9902 (2009).

[b36] Haazen P. P. J. *et al.* Domain wall depinning governed by the spin Hall effect. Nat. Mater. 12, 299–303 (2013). 23377291 10.1038/nmat3553

[b37] Pizzini S. *et al.* Chirality-induced asymmetric magnetic nucleation in Pt/Co/AlO_*x*_ ultrathin microstructures. Phys. Rev. Lett. 113, 047203 (2014). 25105650 10.1103/PhysRevLett.113.047203

[b38] Braun H.-B. Fluctuations and instabilities of ferromagnetic domain-wall pairs in an external magnetic field. Phys. Rev. B 50, 16485 (1994). 10.1103/physrevb.50.16485 9976037

[b39] Kubetzka A., Pietzsch O., Bode M. & Wiesendanger R. Spin-polarized scanning tunneling microscopy study of 360° walls in an external magnetic field. Phys. Rev. B 67, 020401 (2003).

[b40] Hiramatsu R., Kim K.-J., Nakatani Y., Moriyama T. & Ono T. Proposal for quantifying the Dzyaloshinsky-Moriya interaction by domain walls annihilation measurement. Jpn J. Appl. Phys. 53, 108001 (2014).

[b41] Tarasenko S., Stankiewicz A., Tarasenko V. & Ferré J. Bloch wall dynamics in ultrathin ferromagnetic films. J. Magn. Magn. Mater. 189, 19–24 (1998).

[b42] Chapman J. N. The investigation of magnetic domain structures in thin foils by electron microscopy. J Phys. D: Appl. Phys. 17, 623–647 (1984).

[b43] Masseboeuf A., Gatel C., Bayle-Guillemaud P., Marty A. & Toussaint J.-C. Lorentz microscopy mapping for domain wall structure study in L1_0_ FePd thin films. Ultramicroscopy 110, 20–25 (2009). 19766396 10.1016/j.ultramic.2009.08.006

[b44] McVitie S. & Cushley M. Quantitative Fresnel Lorentz microscopy and the transport of intensity equation. Ultramicroscopy 106, 423–431 (2006). 16423466 10.1016/j.ultramic.2005.12.001

[b45] Hubert A. & Schäfer R. Magnetic Domains—The Analysis of Magnetic Microstructures Springer (1998).

[b46] Alejos Ó. & Martnez E. Micromagnetic study of interaction between achiral and homochiral domain walls in ultrathin ferromagnetic strips. J. Appl. Phys. 117, 17D509 (2015).

[b47] Rößler U., Bogdanov A. & Pfleiderer C. Spontaneous skyrmion ground states in magnetic metals. Nature 442, 797–801 (2006). 16915285 10.1038/nature05056

[b48] Jiang W. *et al.* Blowing magnetic skyrmion bubbles. Science 349, 283–286 (2015). 26067256 10.1126/science.aaa1442

[b49] Büttner F. *et al.* Dynamics and inertia of skyrmionic spin structures. Nat. Phys. 11, 225–228 (2015).

[b50] Nagaosa N. & Tokura Y. Topological properties and dynamics of magnetic skyrmions. Nat. Nano 8, 899–911 (2013). 10.1038/nnano.2013.243 24302027

[b51] Fert A., Cros V. & Sampaio J. Skyrmions on the track. Nat. Nano 8, 152–156 (2013). 10.1038/nnano.2013.29 23459548

[b52] Benitez M. *et al.* Engineering magnetic domain-wall structure in permalloy nanowires. Phys. Rev. App. 3, 034008 (2015).

[b53] Vansteenkiste A. *et al.* The design and verification of MuMax3. AIP Adv. 4, 107133 (2014).

[b54] Metaxas P. *et al.* Creep and flow regimes of magnetic domain-wall motion in ultrathin Pt/Co/Pt films with perpendicular anisotropy. Phys. Rev. Lett. 99, 217208 (2007). 18233251 10.1103/PhysRevLett.99.217208

